# Proteomic analysis of protein deposits on worn daily wear silicone hydrogel contact lenses

**Published:** 2008-11-07

**Authors:** Zhenjun Zhao, Xiaojia Wei, Yulina Aliwarga, Nicole A. Carnt, Qian Garrett, Mark D.P. Willcox

**Affiliations:** 1 Institute for Eye Research, Sydney, NSW, Australia; 2 School of Optometry and Vision Science, University of New South Wales, Sydney, NSW, Australia; 3 Vision Cooperative Research Centre, Sydney, NSW, Australia

## Abstract

**Purpose:**

Previous studies have demonstrated deposition of tear proteins onto worn contact lenses. In this study, we used proteomic techniques to analyze the protein deposits extracted from worn daily wear silicone hydrogel contact lenses in combination with different lens care solutions.

**Methods:**

Worn lenses were collected and protein deposits extracted using urea and surfactant. Protein extracts were desalted, concentrated, and then separated using one-dimensional gel electrophoresis. Individual protein components in extracts were identified using liquid chromatography combined with tandem mass spectrometry (LC-MS-MS) after trypsin digestion.

**Results:**

One-dimensional gel electrophoresis revealed that lysozyme and other small proteins (around 20 kDa) were the most abundant proteins in the extracts. LC-MS-MS revealed a wide array of proteins in lens extracts with lysozyme and lipocalin 1 being the most commonly identified in deposit extracts.

**Conclusions:**

Worn contact lenses deposit a wide array of proteins from tear film and other sources. Protein deposit profiles varied and were specific for each contact lens material.

## Introduction

Contact lens wear is an increasingly popular method of vision correction with an estimated 140 million lens wearers worldwide in 2005 [1]. However, due to their direct contact with the eye and tear film, contact lenses can lead to adverse events in the eyes ranging from discomfort to serious infections [2-5]. It has been reported that following insertion, contact lenses adsorb/absorb components from the tear film rapidly and/or progressively depending on lens materials and type of deposit [6-8]. Deposition or contact lens spoilation has been shown to change the physical and/or chemical characteristics of the lens surface [9] and can affect lens clinical performance, contributing to discomfort during lens wear and adverse events [10,11]. Tear film deposition is hypothesized to negatively impact tear film structure and function [12,13]. It has also been proposed that tear film deposits irritate the eye, leading to adverse immunological responses such as giant papillary conjunctivitis [10] and facilitating adhesion and/or growth of bacteria on contact lenses surfaces [14], which would potentially result in microbial keratitis. Although the newly introduced silicone hydrogel contact lenses have been shown to accumulate much less protein deposits than conventional hydrogel lens materials [15] and offer very good clinical performance [16,17], deposit-induced eye problems such as discomfort and giant papillary conjunctivitis are still frequently reported with these contact lenses [18] and are the major reason of discontinuing wear [19].

Products used in lens care regimens or lens care solutions are specifically designed to reduce bacterial colonization and remove deposits from worn contact lenses [20]. Previous reports have demonstrated that the ability of these solutions to remove deposits from silicone hydrogel contact lenses is affected by lens materials [21,22]. Certain contact lens/lens care solution combinations are more effective in deposition reduction. These studies generally investigated the overall amount of deposits on worn contact lenses. The effect of lens care solutions on specific protein deposition profiles on different contact lenses is unknown.

Deposits on worn lenses are mainly proteins and lipids from tear fluid [15,23-27], the residual quantities of which are lens material-dependent and lens care solution-dependent [21]. Due to the small quantity of deposits on most silicone hydrogel lenses, studies have been generally limited to detection of total protein or specific proteins or lipids that bind avidly and in large quantities to particular lens types (i.e., lysozyme to FDA Group IV hydrogel lenses [28]). Since tear fluid comprises many proteins [29], it is quite likely that a variety of proteins will deposit onto contact lenses. Indeed, some low abundant proteins have been detected in contact lens deposits using antibody-based methods [30,31]. A systemic study of the proteome of contact lens deposits using standard proteomic methods, two-dimensional electrophoresis combined with mass spectrometry, is hindered by very low abundance of most proteins in the samples and according to our experience, by the interference of co-extracted lens materials, which causes “smearing” in SDS gels. So far, an extensive systematic proteomic study of the protein species deposited onto worn contact lenses is absent in the literature.

Liquid chromatography combined with tandem mass spectroscopy (LC-MS-MS) is a very sensitive protein identification method for biological samples [32]. It also bypasses a gel separation step and can detect small peptides (<10 kDa) or proteins with very high or very low isoelectric points. Using this technique in the present study, we analyzed protein deposits on four different silicone hydrogel contact lenses in combination with four different lens care solutions.

## Methods

### Contact lenses and lens care solutions

All contact lenses examined were commercially available (Table 1). Worn lenses were obtained from subjects who took part in clinical studies conducted within the Institute for Eye Research (Sydney, Australia). Prior to enrollment, all subjects signed informed consents after the nature and possible consequences of the study were explained. All experimental protocols were reviewed and approved by the Committee for Experimental Research Involving Human Subjects at the Institute for Eye Research and the University of New South Wales (Sydney, Australia) and complied with the Declaration of Helsinki for Experimentation on Humans (1975 and revised in 1983).

**Table 1:** Characteristics of silicone hydrogel lens material used in this study.

**Contact lenses**	Lotrafilcon B	Balafilcon A	Galyfilcon A	Senofilcon A
**Proprietary Name**	O_2_Optix	PureVision	Acuvue Advance	Acuvue Oasys
**Manufacturer**	CIBA Vision	B&L	J&J	J&J
**Water content**	0.33	0.36	0.47	0.38
**Dk**	110	91	60	103
**Surface treatment**	25 nm plasma coating	Plasma oxidation	None	None
**Internal additive**	None	None	PVP	PVP
**FDA group**	I	III	I	I

B&L: Bausch & Lomb, J&J: Johnson & Johnson, Dk: oxygen permeability, PVP: polyvinyl pyrrolidone.

Studies were controlled, prospective, non-randomized, and non-concurrent. Wear schedules were daily wear (DW) with lenses being replaced on a monthly basis, except for senofilcon A lenses with AQuify, ClearCare, or RepleniSH and for galyfilcon A lenses with RepleniSH, which were replaced every two weeks. During lens wear, a lens care solution, also commercially available (Table 2), was used by wearers to rinse lenses for 5 s without rubbing before overnight disinfection. Instruction was given to insert lenses straight from the lens case. Compliance with lens care regime was established at follow up visits. Participants were advised to wear lenses at least five days per week and 6 h per day with no maximum wear time, providing they did not sleep in lenses.

**Table 2:** Components of lens care solutions used in this study.

**Solution**	**Manufacturer**	**Surfactants**	**Preservative**
**ClearCare**	CIBA Vision	Pluronic 17R4	Hydrogen peroxide 3%
**Opti-Free Express**	Alcon	Poloxamine (Tetronic 1304)	Polyquad 0.001%, Aldox 0.0005%
**Opti-Free RepleniSH**	Alcon	TearGlyde (Poloxamine (Tetronic 1304) + C-9 ED3A)	Polyquad 0.001%, Aldox 0.0005%
**AQuify**	CIBA Vision	Sorbitol, Fluronic F127, Dexpanthenol	Polyhexanide 0.0001%

While studies from three protocols over a period of several years were combined, all participants were given the same lens care and wear instructions apart from recommended replacement periods.

To avoid contamination, latex non-powdered surgical gloves were worn when removing contact lenses from patients’ eyes at the completion of lens wear schedule. Worn contact lenses were immediately soaked in 2-3 ml saline solution in clean lens cases to remove any residual tears and stored temporarily at 4 °C. Lenses were separated from saline solution the same day and transferred to −80 °C. The lenses were analyzed within two months of collection.

### Chemicals and reagents

NuPAGE® 4%-12% Bis-Tris 1.0 mm gels and NuPAGE® MES SDS Running buffer were obtained from Invitrogen (Carlsbad, CA). α-cyano-4-hydroxycinnamic acid was purchased from Sigma (Saint Louis, MO). The SYPRO Ruby protein gel stain kit was purchased from Molecular Probes (Eugene, OR). Amicon Ultra centrifugal filter devices (4 ml, cut-off value 5 kDa) were purchased from Millipore (Billerica, MA). Tris, glycerol, glycine, sodium dodecyl sulfate (SDS), and urea were supplied by BDH (Poole, England). Tributyl phosphine (TBP) and acrylamide were purchased from Bio-Rad (Hercules, CA). Bromophenol blue, sequencing grade modified trypsin, and PerfectPure reverse phase C-18 tip were purchased from United States Biochemicals (Cleveland, OH), Promega (Madison, WI), and Eppendorf AG (Hamburg, Germany), respectively.

### Extraction of proteins from worn contact lenses

Proteins accumulated on worn contact lenses were extracted according to the method reported previously [33]. The extraction methods recovered at least 75% of the proteins lysozyme, lactoferrin, and albumin in previous controlled studies [33]. Normally, five lenses from five different patients within a group were pooled to ensure there was sufficient protein in the extracts for LC-MS-MS analysis. However, for balafilcon A lenses, which accumulated more proteins than other lenses [33], one to two lenses each from a single patient were pooled.

### MS identification of protein in SDS gels

For gel separation of proteins, 18 µl of protein extract was first mixed with 6 µl of 4X sample buffer (0.125 M Tris-HCl, 2% SDS, 40% v/v glycerol, 0.8% bromophenol blue, pH 6.8) and incubated at ambient temperature (AT) for 20 min. The extract (20 μl) was loaded onto a NuPAGE® 4%-12% Bis-Tris 1.0 mm mini-gel. The stacking gel contained 4% acrylamide/bis. Electrophoresis was performed at 100 V in NuPAGE® MES SDS running buffer (50 mM MES, 50 mM Tris base, 0.1% SDS, 1 mM EDTA, pH 7.3). Protein bands were visualized by silver staining or in the case where bands were to be identified by mass spectrometry, stained with SYPRO Ruby according to the manufacturer’s instructions.

Protein bands were excised into 1 mm cubes before being transferred into 1.5 ml Eppendorf tubes. Samples were washed with 200 µl 50% acetonitrile/50 mM ammonium bicarbonate for 30 min at 37 °C, dehydrated with 100% acetonitrile, and then dried at 37 °C for 30 min. Utilizing a method described by Herbert and colleagues [34], proteins were reduced and alkylated with 50 µl freshly prepared solution containing 5 mM tributyl phosphine and 10 mM acrylamide in 50 mM ammonium bicarbonate by incubation at AT for 1 h. The samples were washed, dehydrated, and dried again as described above. The samples were digested for 17 h at 37 °C with 50 µl of 6 µg/ml sequencing grade trypsin in 50 mM ammonium bicarbonate. Resulting peptides were extracted twice, each with 100 µl 10% acetonitrile/0.1% trifluoroacetic acid, and sonicated in a water bath for 10 min. Peptide extracts were combined and concentrated to 50 µl in a SpeedVac (Thermo Scientific, Waltham, MA) and then desalted and further concentrated with Eppendorf PerfectPure reverse phase C-18 tip. Finally, peptides were eluted onto a matrix-assisted laser desorption/ionization (MALDI) target plate with 1.5 µl matrix solution containing 8 mg/ml re-crystallized α-cyano-4-hydroxycinnamic acid in 70% acetonitrile/0.1% trifluoroacetic acid. Mass spectrometry (MS) data ranging from 700 to 3500 m/z were acquired automatically with a Waters MicroMass M@LDI (Milford, MA) and calibrated for peptide mass fingerprinting identification using ProteinLynx and MASCOT search engines.

### LC-MS-MS analysis of protein extracts

Protein extracts (10 μl) were digested with trypsin (~200 nM) for 14 h at 37 °C. Resulting peptides were dissolved in formic acid and separated by nano-LC using a Cap-LC autosampler system (Waters, Milford, MA). Samples (5 µl) were concentrated and desalted onto a micro C18 precolumn (500 µm×2 mm, Michrom BioResources, Auburn, CA) with H_2_O:CH_3_CN (98:2, 0.05% heptafluorobutyric acid [HEBA]) at 15 μl/minute. After washing for 4 min, the precolumn was automatically switched (10 port valve; Valco, Houston, TX) into line with a fritless nano column manufactured according to Gatlin [35]. Peptides were eluted using a linear gradient of H_2_O:CH_3_CN (98:2, 0.1% formic acid) to a different H_2_O:CH_3_CN (50:50, 0.1% formic acid) at ~300 nl/min over 30 min. The precolumn was connected via a fused silica capillary (10 cm, 25 µ) to a low volume tee (Upchurch Scientific, Oak Harbor, WA) where high voltage (HV; 2600 V) was applied and the column tip positioned ~1 cm from the Z-spray inlet of a Quadrupole/time-of-flight (QTof) Ultima API hybrid tandem mass spectrometer (Micromass, Manchester, UK). Positive ions were generated by electrospray, and the QTof operated in data dependent acquisition mode (DDA). A Tof MS survey scan was acquired (m/z 350–1700, 1 s), and the two largest multiple-charged ions (counts>20) were sequentially selected by Q1 for MS-MS analysis. Argon was used as the collision gas, and optimum collision energy was chosen (based on charge state and mass). Tandem mass spectra were accumulated for up to 8 s (m/z 50–2000). Peak lists were generated by MassLynx (version 4.0 SP1, Micromass) using the Mass Measure program and submitted to the database search program, MASCOT (version 2.1, Matrix Science, London, England). Search parameters were precursor and product ion tolerances ±0.25 Da and 0.2 Da, respectively; Met(O) specified as variable modification, enzyme specificity was trypsin, one missed cleavage was possible and the non-redundant protein sequence database in NCBInr (July 2007) searched. For protein identification, the p value (probability that the observed match is a random event) was set at p≤0.05.

### Statistical analysis of data

To test for differences in the total number of protein types or number of specific protein groups that were extracted from the same lens type after use of different lens care systems or same lens care system using different lenses, the proportions test was used with significance set at p<0.05.

## Results

### Identification of gel bands

**Figure 1:**
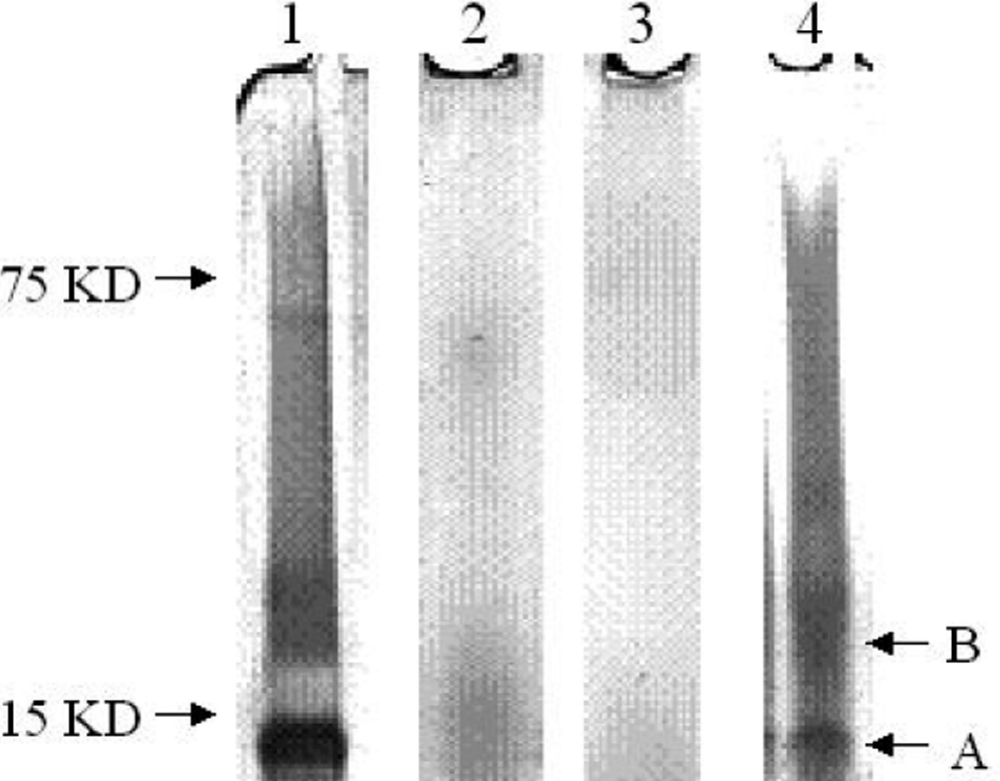
Examples of SDS gel analysis of the protein extracts. Lane 1 shows the balafilcon A lens with Opti-Free extract. Lane 2 shows the lotrafilcon B lens with ClearCare extract, and lane 3 shows the senofilcon A lens with ClearCare extract. Lane 4 shows galyfilcon A lens with Opti-Free extract. Band A is the tear lysozyme, and Band B shows unidentified proteins.

In an attempt to identify individual proteins in the protein extracts, samples were separated using 4%-12% SDS gels and silver-stained. A single band around 14-15 kDa and another much broader band at around 20 kDa were observed in samples that exhibited high total protein (~5 µg/ml). However, results were not easy to analyze due to smearing on the gels (Figure 1). The band profile was the same for all the samples. The two major bands were cut from the gel and analyzed using MS following trypsin digestion. The 14-15 kDa band was identified as lysozyme, but no identification could be made for the band at 20 kDa. Therefore, an alternative approach using total tryptic digestion of extracts followed by LC-MS-MS was investigated.

### LC-MS-MS analysis of the extracts

Proteins detected by LC-MS-MS are listed in Appendix 1. Sixty-eight proteins were identified from the samples. The most frequently detected proteins among the 16 samples were lysozyme (14/16; MW: 14.8 kDa; PI: 10.4), lipocalin (13/16; MW: 19.2 kDa; PI: 4.8), secretory immunoglobulin A (sIgA) fragments (12/16), proline rich 4 protein (11/16; MW: 15.1 kDa; PI: 7.0), keratin 1 (11/16; MW: 66.0 kDa; PI: 4.9), lactoferrin (10/16; MW: 78.0 kDa; PI: 8.7), keratin 10 (9/16; MW: 59.5 kDa; PI: 6.2), retinoic acid receptor responder 1 (9/16; MW: 33.3 kDa; PI: 8.7), and heparan sulfate proteoglycan (8/16; MW: 468.8 kDa; PI: 6.0).

The detection rate of immunoglobins was significantly lower in lotrafilcon B lens extracts than that in extracts from other lens types (p<0.05). With the exception of albumin in the AQuify group, albumin, hemoglobins, and complement proteins were not recovered from the lotrafilcon B or the balafilcon A lenses. However, heparin sulfate proteoglycan and DMBT-1 (a mucin-like glycoprotein) were recovered from these lens types more often compared to other lenses.

For lotrafilcon B lenses, use of ClearCare significantly reduced the number of detected proteins when compared with use of Opti-Free RepleniSH (p<0.01). No other significant differences in the number of detected proteins for lotrafilcon B lenses were found. For galyfilcon A lenses, use of Opti-Free RepleniSH or ClearCare reduced the number of detected proteins when compared with use of Opti-Free Express or AQuify (p<0.02). In the case of senofilcon A lenses, Opti-Free Express and ClearCare groups had fewer protein species than RepleniSH or AQuify groups (p < 0.01). Similarly fewer proteins were extracted from balafilcon A lenses in the Opti-Free Express and ClearCare groups compared to the RepleniSH or AQuify groups (p < 0.05). For a given lens care solution, significant differences in the number of proteins recovered was found between different lens types using Opti-Free Express (galyfilcon A>lotrafilcon B>senofilcon A=balafilcon A). In the AQuify group, the number of detected proteins in galyfilcon A and senofilcon A lenses was significantly higher than in lotrafilcon B lenses (p<0.05).

## Discussion

Silicone hydrogel lenses are becoming the lens of choice for many contact lens wearers [16,36]. Although superior in terms of oxygen permeability compared with poly HEMA-based lenses, silicone hydrogels lenses are still not problem-free [18]. A significant proportion of contact lens wearers still experience ocular discomfort, inflammation, and infection with these lenses [1,19]. It has been estimated that 80% of clinical problems and 30% of aftercare visits relating to wearing extended wear of conventional contact lenses may be attributed to deposition of tear-derived substances on the lens surface [37,38]. However, certain deposits may have beneficial effects [9]. Development of new lens materials that only adsorb/absorb beneficial substances and not harmful deposits or an easy-use lens care solution that can effectively clean worn contact lenses may eliminate many of the contact lens related eye complaints and increase comfort for wearers.

The current study reports results of proteomic analyses of protein deposits extracted from different silicone hydrogel contact lenses when used in combination with various lens care solutions. Deposition (adsorption and/or absorption) of proteins onto contact lenses may involve surface adsorption and penetration of small molecules into the lens matrix [26,27,39,40]. The deposition process is not completely understood but is known to be affected by several factors such as lens material water content [28,41], surface hydrophilicity [42], surface charge [28,43,44], and tear film characteristics of an individual subject [6,11] as well as interactions among various deposits on the lens surface [45].

The efficacy of protein extraction from the surface of all lenses used in the current study has been tested [33]. For major tear proteins (lysozyme, lactoferrin, and albumin), extraction efficacy ranged from 76.2% for albumin from balafilcon A lenses to 95.7% for lysozyme from galyfilcon A lenses. However, given the range of proteins found in the current study, it is possible that a given protein may have a special affinity to a specific lens material, and therefore, we cannot conclude that the lack of protein extracted from a certain lens/solution combination is not due to this process.

Of the silicone hydrogel lens types examined in the current study, balafilcon A is classified as an FDA group III lens (ionic, low water content) and has been found to accumulate significantly greater amounts of protein deposits after DW than other lenses [33]. To achieve a wettable surface, the lenses are treated by plasma oxidation producing hydrophilic glassy islands [46]. Silicate islands do not completely occlude the surface, resulting in a non-uniform surface divided into hydrophobic and hydrophilic areas. It is possible that this characteristic allows greater deposition of proteins. The negative charge of the polymer (due to the acidic carboxyl group in N-vinyl amino acid) would be expected to attract positively charged proteins such as lysozyme [47]. Indeed, the ionic matrix is prone to accumulating protein deposits, especially lysozyme [26,28,44]. Results in this paper reveal that the number of detected protein species in extracts from this lens type is not higher than the numbers found from other lenses, indicating that the greater deposition seen with balafilcon A lenses may be a general phenomenon and not a result from accumulation of a particular protein species.

Distinct from balafilcon A lenses, the lotrafilcon B, galyfilcon A, and senofilcon A lenses are manufactured from FDA group I materials (non-ionic, low water content). The surface of lotrafilcon B lenses is plasma-coated whereas galyfilcon A and senofilcon A are free of surface coating. Galyfilcon A and senofilcon A lenses contain an internal additive, polyvinyl pyrrolidone (PVP). The PVP may account for the common character of the two lenses to attract albumin, hemoglobins, and complement proteins and repel proteoglycans and mucins. The mechanism of the difference in attracting different proteins by different lens materials and surface treatments and their clinical implications warrant further studies.

Components of lens care solutions may change the chemical and/or physical characteristics of the lens surface and/or stick to the surface or penetrate into the lens matrix. Indeed, the tested solutions affected the numbers of proteins detected for certain contact lens types, but their effects were dependent on lens materials. For example, Opti-Free Express reduced the number of detected proteins in extracts of senofilcon A and balafilcon A lenses compared to the overall number of proteins detected in galyfilcon A lenses. The mechanism is unknown and also warrants further study to develop a better lens care product.

Among the proteins identified in this paper, some have been detected in lens deposits by other researchers. Using different methods and extracts from various contact lenses, lysozyme [15,48-50], lipocalin [50,51], lactoferrin [50,52], IgG, sIgA, IgM, complement C3 [53,54], IgE [55], secretory phospholipase A2 [56], albumin [8,50], and vitronectin [31] have been found to deposit onto various lens materials. Interestingly, keratins 1 and 10, retinoic acid receptor responder 1, and heparan sulfate proteoglycan were frequently detected in the current study but not reported by others previously. The clinical significances of these deposits are unknown at present but warrant further studies.

A recent publication by Green-Church and Nichols reported the first attempt to analyze contact lens deposits using LC-MS-MS [50]. Less than 20 protein species were identified in this paper. We detected many more proteins (total 68) including some of the proteins detected in the previous study [50]. Green-Church and Nichols only examined galyfilcon A or lotrafilcon B in combination with either AQuify or ReNu with MoistureLoc (Bausch and Lomb, Rochester, NY). When comparing the galyfilcon A or lotrafilcon B lenses that were used with AQuify to the results of the current investigation, the major differences are in the number of proteins identified (higher in the current investigation) and the presence of lacritin and secretoglobins (mammaglobins), which were not identified in these particular lens/solution combinations in the present investigation. The Green-Church/Nichols study [50] did not wash the lenses after wear and analyzed lenses directly from the eye. We have noted that if there is no washing before the extraction of proteins from lenses, the extract appears to be very similar to the tear film (unpublished results). Perhaps this is one reason for the differences noted between the two studies. Also, the method of protein extraction from lenses was different between the two studies, which may also affect protein species recovered. In the current study, some of the proteins detected were not tear proteins such as keratins, which made up a significant proportion of the 68 protein types identified. These proteins may originate from the skin of the fingers or surrounding of the eyes that come into contact with contact lenses when the participants perform their daily lens care procedure and lens insertion.

The clinical trials from which the tested worn lenses were collected were not designed solely for the deposit analysis. They had to meet other study requirements so the wear lengths of these lenses were not the same (one month or two weeks, see Methods). It has been documented that the accumulated amount of some deposits is wear length dependent [6,7]. Different wear times would not impinge on the data analysis in the present study since this was a qualitative proteomic analysis and did not attempt quantification of the proteins identified. However, some care should be taken when comparing the results between lenses worn for different time periods.

Due to the use of pooled samples, the results in this study do not give any information about variation between patients within a group. Previous reports have demonstrated that deposit accumulation on worn contact lenses is affected by tear film characteristics of individual subjects [6,11]. Thus, it is possible that patient-to-patient variability exists within the current study. On the other hand, the use of pooled samples should reduce the component of patient-to-patient variation and reveal overall differences between lens/solution groups. The use of lesser numbers of pooled lenses in the balafilcon A lens groups is a disadvantage in this aspect. The influence of patient-to-patient variation will be addressed in subsequent research.

In conclusion, this study has shown that silicone hydrogel lenses can adsorb/absorb proteins from the tear film and other sources. The accumulated deposition of individual protein types was dependent on the polymer materials from which the lenses had been made and lens care solutions used with the lenses.

## Appendix 1. List of proteins identified in the protein extracts by LC-MS-MS.

To access the data, click or select the words “Appendix 1.” This will initiate the download of a compressed (pdf) archive that contains the file.
